# Supplementary material for: Design strategies for tourism cultural and creative products of Hetuala city based on KANO and AHP
Source: PLoS One. 2025 Dec 9;20(12):e0338573. doi: 10.1371/journal.pone.0338573 (PMC12688142; doi:10.1371/journal.pone.0338573)
Supplement: Attachment — Submitted filename: Writing the review PLOS ONE Penultimate 2.docx [file pone.0338573.s001.docx]

**The assessment of the need to design cultural and creative products based on the KANO and AHP Model： The  Case Study of Hetuala City**

**Writing the review**

The purpose of the review is to provide the editors with an expert opinion regarding the validity and quality of the manuscript under consideration. The review should also supply authors with explicit feedback on how to improve their papers so that they will be acceptable for publication in *PLOS ONE*. As you write, consider the following points:

**What are the central claims of the paper, and how significant are they for the discipline?**

The paper examines how to enhance the design and development of cultural and creative products in Hautala City, located in the Manchu Autonomous County of Liaoning Province, China. This was effectively achieved through the application of the KANO and AHP models in analysing the demand for, or otherwise, creative and cultural products. The claims are significant because they open another vista in analysing the effectiveness of the demand for innovative and artistic products by local consumers and tourists.

**Are the claims properly placed in the context of the previous literature? Have the authors treated the literature fairly?**

The literature review section could be enhanced by assigning it its own heading, rather than merging it with the introduction. Additionally, some parts of the analysis could be incorporated into the literature review to enhance clarity and logical coherence. This change would help position the analysis as a distinct component of the work. Properly citing previous literature will also contribute to the advancement of this project. Furthermore, the differences and relationships between the KANO model and the AHP (Analytic Hierarchy Process) should be examined in relation to the topic.

**Do the data and analyses fully support the claims? If not, what other evidence is required?**

The data presented supports the claims (though overtly quantitative) and fits into the study to drive the research theme. The method used does not require supporting information, as it is explicitly stated in the text.

**If the paper is considered unsuitable for publication in its present form, does the study itself show sufficient potential that the authors should be encouraged to resubmit a revised version?**

With minor structural corrections, the text has enough potential for publication. The author should be encouraged to make corrections and resubmit for publication.

**Are original data deposited in appropriate repositories and accession/version numbers provided for genes, proteins, mutants, diseases, etc.?**

I am not sure about this.

**Does the study conform to any** [**relevant guidelines**](https://journals.plos.org/plosone/s/best-practices-in-research-reporting#loc-reporting-guidelines-for-specific-research-resources-and-study-types) **such as CONSORT, MIAME, QUORUM, STROBE, and the** [**Fort Lauderdale agreement**](https://www.sanger.ac.uk/wp-content/uploads/fortlauderdalereport.pdf)**?**

I am not sure about this.

**Are details of the methodology sufficient to allow the experiments to be reproduced?**

Yes, because they are tested and explicit. Many publications testify to that. The method is replicable in varied circumstances.

**Is any software created by the authors freely available?**

Not applicable.

**Is the manuscript well organized and written enough to be accessible to non-specialists?**

The logical arrangement of the text could be improved to follow a particular order used in academic research, such as the IMRAD method, which logically captures all the sections of the research. The methods and analysis sections are overtly quantitative; consequently, it may be challenging for a non-expert to interpret the data correctly.

**Is it your opinion that this manuscript contains an** [**NIH-defined experiment of Dual Use concern**](https://grants.nih.gov/grants/guide/notice-files/NOT-OD-13-107.html)**?**

This is not applicable here.

**Other points to note**

**1. Title and Abstract**

Although the abstract is professionally written, it could be improved for better clarity. First, the introduction should be condensed to one or two sentences that clearly convey the essence of the study.

It is essential to include a description of the population sample, as well as to provide a clear explanation of acronyms such as KANO and AUP.

Additionally, the study's results should be presented more explicitly. The methodology needs to be communicated more transparently, using clear and accessible language that is easy for readers to understand.

The word count should be limited to 250-300 words for a more concise abstract.

The title could also be shortened to 15 words. (APA 7^th^ edition)

**2. Research Background and Objectives**

There are a few instances of repetitive ideas and phrases.

Similar paragraphs should be merged or connected with linking words to ensure a smooth flow of ideas.

Connecting words should be appropriately placed; for instance, 'finally' is repeated multiple times.

For clarity, objectives should be presented in bullet points.

**3. Research Scope and Methods**

This section needs a thorough review to reduce the content to summaries

In-text citation should indicate APA 7th edition

Certain sections in the introduction would benefit from being made more explicit with the addition of diagrams and illustrations.

**4. Theories and Models**

The explication of the acronym KANO is required.

For clarity, the highlights of KANO should be put in bullet points

The relationship between the two models and how they drive the study should be analysed.

**6. Findings**

This section could be indicated as such in a separate heading.

**7. Discussion**

Some of the ideas, especially those related to the history of the creative city, should be presented in the introduction and literature review for logical coherence.

Some itemised issues could be discussed as a whole, e.g., contents like 1st, 2nd, 3rd, etc., for a free flow of understanding.

**8**. **The Conclusion and Recommendations**

This section should be written separately

**9. Reference**

References should follow APA 7th edition.( If that is the requirement)

Although confidential comments to the editors are respected, any remarks that might help to strengthen the paper should be directed to the authors themselves.
