# Supplementary material for: Design strategies for tourism cultural and creative products of Hetuala city based on KANO and AHP
Source: PLoS One. 2025 Dec 9;20(12):e0338573. doi: 10.1371/journal.pone.0338573 (PMC12688142; doi:10.1371/journal.pone.0338573)
Supplement: Attachment — Submitted filename: Response to Reviewers.docx [file pone.0338573.s002.docx]

Dear Academic Editor and Reviewers：

We sincerely thank you for your thorough review of our manuscript and for your valuable comments. Your suggestions have been extremely helpful in improving the quality of our research and the manuscript. In response to the reviewers’ feedback, we have thoroughly revised the manuscript. The main modifications are summarized as follows:

**1.**Manuscript Structure Adjustment:**** The overall structure of the manuscript has been reorganized into five sections: Introduction, Literature Review, Methodology, Results and Discussion, and Conclusion and Recommendations.

**（1）Literature Review:** A Literature Review section has been added, providing conceptual definitions and explanations for “tourism cultural and creative products,” the KANO model, and the AHP method, along with relevant studies by domestic and international scholars. In addition, the differences and connections between KANO and AHP have been clarified to help readers better understand the theoretical foundation of the study and the rationale for the chosen methodology.

**（2）Methodology:** A dedicated Methodology section has been added, providing a detailed description of the research design, implementation process, and analytical steps. The specific application procedures and operational details of the KANO and AHP methods have been clearly explained, enhancing both transparency and methodological rigor.

**（3）Results and Discussion:** The Results section has been reorganized and structured. In the Discussion section, comparisons and interpretations based on KANO and AHP analysis results have been added, ensuring that the research findings directly address the research questions.

**（4）Conclusion and Recommendations:** A Recommendations subsection has been added. Based on the research findings, specific suggestions for the design and development of tourism cultural and creative products have been provided to enhance the practical applicability and relevance of the study.

**2.Title and Abstract Revision:** The manuscript title and abstract have been revised. The new abstract has been rewritten in concise and clear academic language, highlighting the research objectives, methods, and key conclusions, ensuring that it accurately reflects the overall scope and contribution of the study.

**3.Citations and Reference Formatting:** All in-text citations and references have been thoroughly checked and standardized according to APA 7th edition guidelines. Some references have been updated or supplemented to better connect the study with existing scholarly work, thereby improving the manuscript’s academic rigor and completeness.

**Response to the Academic Editor**

**1.**Funding Statement:**** The funding statement has been revised and is now provided in detail within the cover letter.

**2.**Ethics Statement:**** The ethics statement has been moved to the Methods section of the paper.

**3.**Conceptual Definitions:**** A dedicated section in the Literature Review has been added to define the concepts of “tourism cultural and creative products,” the KANO model, and the AHP method. Relevant references have been included to support these definitions, thereby strengthening the theoretical foundation of the study.

**4.**Methodology:**** A separate Methodology section has been added, providing detailed descriptions of the research process, steps, and implementation procedures to improve transparency and reproducibility.

**5.**Results:**** The Results section has been revised to provide a systematic analysis based on the findings from the KANO and AHP models, ensuring that the discussion is more data-driven and logically consistent.

**6.**In-text Citations and Reference Format:**** All in-text citations and the reference list have been thoroughly checked and revised to ensure full compliance with the journal’s formatting requirements.

**7.Data availability statement:** All relevant data are within the manuscript.

**Response to the Reviewer #1**

**1.**Literature Review:**** We have added a dedicated Literature Review section, which defines the concept of “tourism cultural and creative products” and illustrates, with examples, their role in promoting regional economic development. Additionally, the Introduction section has been updated to summarize the current design and sales status of tourism cultural and creative products in Hetu Ala City, enhancing the clarity and completeness of the research background.

**2.**Language Consistency:**** All references to “tourism cultural and creative products” throughout the manuscript have been standardized to ensure consistent language style, improving readability and overall professionalism.

**3.**Abstract:**** The Abstract has been revised to provide a detailed description of the research objectives, methods, results, and conclusions, making it more comprehensive and structured, and enabling readers to quickly grasp the core content of the study.

**4.**Methodology:**** A dedicated Methodology section has been added, providing detailed descriptions of the research design, implementation process, and analytical steps. The specific application procedures and operational details of the KANO and AHP methods have been clearly explained to enhance the transparency and rigor of the methodology.

**5. **Research Results:**** The Results section has been reorganized and structured. The findings from the KANO and AHP analyses are compared and interpreted systematically, making the research outcomes more clearly aligned with the research questions.

**6.**Interviewee Selection:**** Interview participants were selected based on stakeholder relevance, including tourists, cultural and creative product sales personnel, consumers, and professionals in the design industry. The tourist sample covers a range of occupations (e.g., villagers, students, teachers, workers), while in-depth interviews focused on more experienced sales personnel, consumers, and design industry professionals to obtain more precise and professional insights into consumer needs.

**7.**References:**** Citations have been revised for sections defining concepts and presenting arguments to ensure that all claims are properly supported. The reference format has also been adjusted to comply with the journal’s guidelines.

**Response to the Reviewer #2**

**1.**Manuscript Structure Adjustment:**** We have optimized and revised the overall structure of the manuscript. The revised structure now includes Introduction, Literature Review, Methodology, Results and Discussion, and Conclusion and Recommendations, which improves the clarity of the research logic and enhances the overall organization.

**2. **In-text Citations and Reference Format:**** We have thoroughly revised all in-text citations and the reference list to ensure full compliance with the journal’s formatting requirements.

**3.**Discussion Section Revision:**** The Discussion section has been reorganized by integrating its original content with the Literature Review. This makes the discussion more systematic and strengthens the connection between our research findings and existing literature, thereby enhancing the rigor and scholarly value of the arguments.
